# Children’s Privilege in COVID-19: The Protective Role of the Juvenile Lung Morphometry and Ventilatory Pattern on Airborne SARS-CoV-2 Transmission to Respiratory Epithelial Barriers and Disease Severity

**DOI:** 10.3390/biomedicines9101414

**Published:** 2021-10-08

**Authors:** Norbert Hofstätter, Sabine Hofer, Albert Duschl, Martin Himly

**Affiliations:** Department of Biosciences, Paris Lodron University of Salzburg (PLUS), 5020 Salzburg, Austria; norbert.hofstaetter@plus.ac.at (N.H.); sabine.hofer@plus.ac.at (S.H.); albert.duschl@plus.ac.at (A.D.)

**Keywords:** aerosol, alveolar–interstitial region, droplets, pathogenesis, pathophysiology, pediatric, pneumonia, risk factor

## Abstract

The incidence of severe COVID-19 in children is low, and underlying mechanisms for lower SARS-CoV-2 susceptibility and self-limiting disease severity are poorly understood. Severe clinical manifestations in adults require SARS-CoV-2 inoculation in the lower respiratory tract, establishing a pulmonary disease phase. This may be either accomplished by direct inoculation of the thoracic region upon exposure to virion-laden aerosols, or by infection of the upper respiratory system and aspiration of virion-laden aerosols originating right there into the lower respiratory tract. The particularities of epithelial barriers as the anatomical site of first viral deposition specifically determine the initial characteristics of an innate immune response, emerging respiratory tissue damage and dysfunctionality, and hence, severity of clinical symptoms. We, thus, investigated by in silico modeling whether the combined effect of juvenile lung morphometry, children’s ventilatory pattern and the peculiarities of the virion-laden aerosols’ properties, render children more resilient to aerosol deposition in the lower respiratory tract. Our study presents evidence for major age-dependent differences of the regional virion-laden aerosol deposition. We identified deposition hotspots in the alveolar–interstitial region of the young adult. Our data reveal that children are void of corresponding hotspots. The inoculum quantum in the alveolar–interstitial region hotspots is found to be considerably related to age. Our results suggest that children are intrinsically protected against SARS-CoV-2 inoculation in the lower respiratory tract, which may help to explain the lower risk of severe clinical manifestations associated with a pulmonary phase.

## 1. Introduction

Identifying factors driving the transmission and infectivity of the severe acute respiratory syndrome coronavirus 2 (SARS-CoV-2) is critical for making the right choice among possible infection control and containment measures. Each wavelike resurgence of coronavirus disease 2019 (COVID-19) pandemic has reawakened the intense debate about the particular role of children and young adults in SARS-CoV-2 transmission. The incidence of severe COVID-19 in children is low, although there is ample evidence that seroconversion rates of children and adults are not different [1,2,3]. In a cohort of 742 pediatric patients seeking medical advice in a specialized children’s hospital and being tested SARS-CoV-2-positive during routine testing, with a median age of 6 years, 21% (156/742) presented with upper respiratory tract (URT) symptoms and only 3.9% (29/742) with lower respiratory tract (LRT) involvement [4]. This disparity may be co-founded by more than one factor. It may include different expression patterns of viral docking receptors, a pre-activated innate immunity, limiting SARS-CoV-2 replication and COVID-19 disease progression in children at an early stage [5], or differences in the predominant route of transmission and anatomical sites of first inoculation.

As established by the World Health Organization’s COVID-19 Clinical management: living guidance (WHO/2019-mCoV/clinical/2021.1), the clinical spectrum of COVID-19 in adults with moderate, severe or critical manifestations is entirely linked to a pulmonary phase. Thus, deposition of a disease-initiating SARS-CoV-2 dose in the thoracic region is a key event. Direct inoculation at the LRT may result from exposure of susceptible individuals to virion-laden respiratory aerosols with a high probability of depositing at hotspots in the secondary pulmonary lobules of the peripheral lung upon inhalation, which we already confirmed for adults in an earlier study [6]. Alternatively, infection of epithelia of the oral-nasal cavity, the pharynx, and larynx may propagate, upon self-aspiration of virion-laden aerosols originating right there, into the LRT. The mechanisms of aerosol generation and anatomical sites of origination in the URT and LRT are well described [7]. Among these, the most promising mechanism providing virion-laden aerosols for aspiration is fluid film burst and shear-induced surface instability in the airway lining fluid at the larynx during vocalization, because aerosols may be trapped in the residual URT volume at the end of the expiratory phase. Thus, virus-laden aerosols are available for immediate aspiration at the beginning of a new breathing cycle. Both coexisting scenarios, direct inoculation in the LRT and SARS-CoV-2 propagation by aspiration subsequent to viral replication in the URT, link aerosol transmission or displacement to LRT deposition of virion-laden aerosols, hence, to a pulmonary phase of COVID-19 and more severe disease manifestations associated with higher disease burden.

The general sentiment on pathogen-induced lung infections emphasizes that the primary site of inoculation is the URT and a stepwise migration via conducting airways towards the distal regions of the LRT has been proposed [8,9]. Although this might be true for fomite or large droplet-transmitted pathogens, it can be challenged for the pathogen-laden aerosol transmission route. From the perspective of an immunologist, reversing this approach would come along with a major implication on pathogenesis and disease severity. Initial replication of SARS-CoV-2 on epithelia of the URT would be without severe clinical manifestations, but viral antigen exposure right there would allow bridging the innate immune response to an adaptive immune response and, with some delay, effectively preventing viral replication in the LRT and mitigating the stepwise SARS-CoV-2 propagation to the smallest functional units, the second pulmonary lobules. These are the anatomical sites of chest CTs anomalies in the peripheral lungs in early phase COVID-19 pneumonia. In contrast, without the head start of an activated innate immunity in the URT, initial deposition and replication of SARS-CoV-2 on epithelia in the most vulnerable regions of the LRT, the alveolar–interstitial region, is neither effectively hindered by physical or soluble barriers or by clearance through the limited number of resident alveolar macrophages in early disease. Hence, initial viral damage to pneumocytes and the inflammatory immune response to pathogen- and danger-associated molecular patterns will induce alveolar–interstitial edema, alveolar filling and collapse, recruitment of neutrophils and monocytes and will finally compromise oxygenation, all before an arising adaptive immune response can regain control on viral replication.

Children’s lungs are not just miniaturizations of adult lungs. Thus, the anatomy and physiology of breathing might involve a very different regional selectivity for the retainment and deposition of virion-laden aerosols compared to an adult’s respiratory tract. A dissimilar risk of LRT disease and COVID-19 severity upon aerosol transmission, given identical exposure conditions, should be expected. A child, in comparison to an adult, has an increased basal metabolic demand, which has to be matched by an increased respiratory turnover, relative to the body mass [10]. Hence, children were found to be more susceptible for developing adverse pulmonary effects upon inhalation of urban air pollutants [11], of toxic gaseous substances, or of respirable particulate matter [12,13], which could include fungal spores, bacteria and possibly virions. These arguments should hold true for airborne transmission and thoracic deposition of SARS-CoV-2-laden aerosols, but this notion is not supported by the observed rare evidence of LRT clinical manifestations in children, although the role of aerosol transmission [14] and the particular implications for the etiology of COVID-19 is increasingly acknowledged as the major route for SARS-CoV-2 spreading in crowded and confined spaces [15]. 

A minimum infectious dose for SARS-CoV-2 is not established. However, most recent studies, using deep sequencing of the SARS-CoV-2 genome variability and considering intra-host evolution during human-to-human transmission, elucidated a very narrow genomic transmission bottleneck by investigating the mutational SARS-CoV-2 diversity in confirmed infector–infectee pairs [16,17,18]. These studies report a bottleneck-size of one to eight virions in validated transmission events, which is of major importance for understanding SARS-CoV-2 transmission dynamics. Low disease-initiating doses favor a high selectivity of different anatomical regions of the respiratory tract for initial viral replication, caused by inequalities of virion-laden aerosol deposition probabilities. This implies a strong predilection of aerosol deposition hotspots, which might be a result of age-specific lung morphometry or pre-existing conditions.

We, thus, investigated, by in silico modeling of a preschool child at the age of three (3y-child), a child at the age of eight (8y-child), and an adult at the age of 21 (21y-adult), the age disparity of initial virion-laden aerosol deposition resulting from (i) differences in the lung morphometry, (ii) differences in the ventilatory pattern, and (iii) physiochemical properties of ambient air virion-laden aerosols. To mimic a realistic exposure situation, we applied virion-laden ambient air aerosols, originating from breathing and vocalization of infected individuals. We aimed to identify age-specific variations in SARS-CoV-2 susceptibility to airborne transmission, co-founding the differences in the frequency of a pulmonary COVID-19 phase between children and adults, thus, in COVID-19 pathogenesis and disease severity. 

## 2. Materials and Methods

A combined in silico simulation and data analysis approach was performed. First, the deposition probability of ambient air and respiratory virion-laden particles (VLPs) in the size range between 0.3–100 µm was determined for the thoracic region. We considered the lung morphometry of a 3y-child, an 8y-child, and a 21y-adult. Second, deposition heatmaps, with detailed data on all lung generations, were established. Third, VLPs originating from specific expiratory activity, vocalization and breathing, most relevant for indoor aerosol transmission, were investigated. Particular aerosol size distributions reflecting these expiratory maneuvers were used to establish lung lobe-, lung generation-, and age-specific VLP deposition heatmaps.

### 2.1. Ambient Air Respiratory Aerosol Particle Modeling

For establishing the deposition probability data by in silico modeling the physicochemical properties of VLPs and an age-specific anatomical regions and airway generation model were defined. For investigation of virion-laden aerosol deposition the size range lower bound for VLPs was set at 0.3 µm, because the probability of virion adsorption to smaller particles is negligible. The airborne time for VLPs with a size larger than 100 µm, the selected upper bound, is limited due to gravitational settling; thus, these will not contribute to long-distance aerosol transmission. For our modeling purpose, a VLP is composed of evaporated mucus remnant in equilibrium with ambient air, relative humidity lower than 60% and room temperature, considering an evaporation shrinkage factor of 0.5, according to Nicas et al. [19], and Holmgren et al. [20], and with one single virion adsorbed. In the moment of exhalation, respiratory aerosol particles are in an evaporation equilibrium with the breath cloud (90% relative humidity, 28 °C) [21]. For particles with a size smaller than 20 µm a transition to a new evaporation equilibrium with ambient air conditions takes place in the order of milliseconds to tenths of seconds [7,22]. This single virion approach is well supported by literature which reports 10^6^ to 10^11^ viral RNAs per milliliter [23]. Following aerosolization and random adsorption of virions and using the highest concentration, most respiratory aerosol particles in the low µm size range contain just one virion or are void of virions. This observation was long before confirmed by Couch et al. [24] in the context of adenoviruses, where the vast majority had only one virion and this broadly concurs with Poon et al. [25] and Stadnytskyi et al. [16]. The mucus and VLP composition and the thereof derived density, before and after evaporation, was adopted from Hofer et al. [6] to be 1.04 g/mL and 1.3 g/mL, respectively. SARS-CoV-2 virion diameter was defined at 0.12 µm and the density at 1.2 g/cm^3^ [26].

### 2.2. Deposition Simulation and Lung Modeling

VLP deposition was determined using the Multiple Path Particle Model (MPPD V3.04; Applied Research Associates, Inc., Huntsville, AL, USA) [27,28] and the age-specific 5-lobe lung model from Mortensen [29,30,31]. MPPD supports in silico aerosol deposition modeling in the human lung, based on numerical calculations of equations considering different breathing models, governing particokinetics and particle deposition, physiological parameters of the lung, and physicochemical properties of aerosols under investigation.

**Figure 1:**
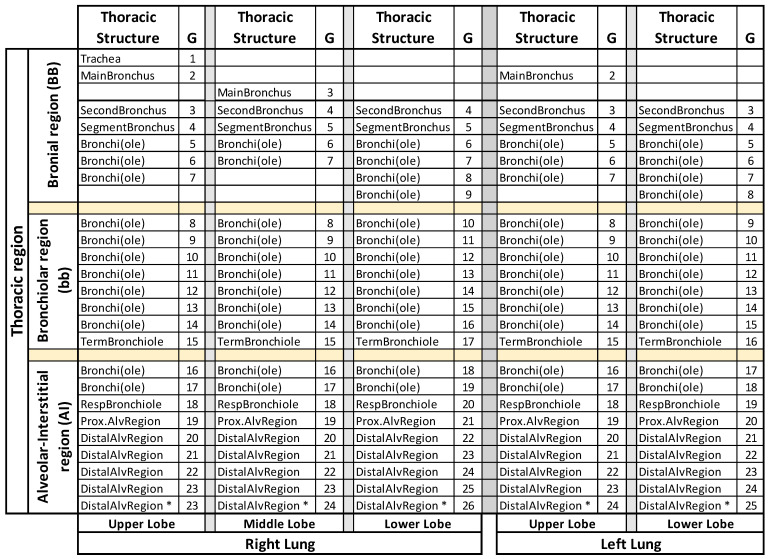
Anatomical sub-structures of the thoracic region according to the Mortensen 5-lobe model applied in the VLP deposition simulation. Common structures are indicated on the far left. G, generation number. * generation absent in 3y-child lung model. VLP, respiratory virion-laden particle.

Mortensen relies on lung morphometry data derived from human lung casts of individuals at different age. The model represents an asymmetric human lung with five lobes. In brief, we divided the respiratory system into four anatomical regions: the extrathoracic region, the bronchial (BB) region, the bronchiolar (bb) region, and the alveolar–interstitial (AI) region [32], the latter three compose the thoracic region. Using this model of the respiratory tract, we compartmentalized the thoracic region in up to 115 sub-regions; segmentation starts with the common anatomical region of the trachea and expands to the most distal alveolar region of each individual lobe of the lungs (Figure 1).

**Figure 2:**
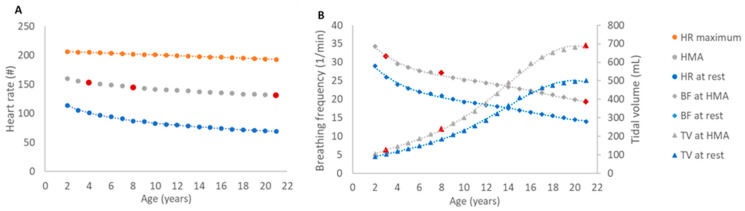
Determination of age-specific respiratory parameters for a moderate to marked activity level. The age-specific respiratory parameters “tidal volume” and “breathing frequency” for moderate to marked activity levels were calculated based on ventilation-perfusion matching. (**A**) The determination of the heart rate for a half-maximal activity level (HMA) was calculated midmost of heart rate maximum and heart rate at rest. (**B**) Breathing frequency and tidal volume at HMA were calculated by adjusting the age group-specific values at rest with the relative increase of heart rate from rest to HMA to achieve a match of the respiratory minute volume. In this simplified model the relative increase was split evenly between breathing frequency and tidal volume. HMAs, breathing frequency and tidal volume for the three age groups (3y-child, 8y-child, 21y-adult) applied in the in silico modeling are marked in red. HR, heart rate; HMA, heart rate for a half-maximal activity level; BF, breathing frequency; TV, tidal volume.

For modeling a comparable activity level in all three age groups, reflecting moderate to marked physical activity, we established age-specific breathing frequencies and tidal volumes. Based on the principle of the ventilation-perfusion matching at any activity level, we first determined a heart rate for a half-maximal activity level (HMA) for each age group (Figure 2A). The heart rate at rest was derived from established data by extrapolating to the age of 19 to 21 years [33]. The maximum heart rate was calculated with the formula of Tanaka et al. [34]. HMA was calculated midmost of heart rate maximum and heart rate at rest. Subsequently, the relative increase of heart rate at rest, necessary to achieve the HMA, was used to adjust the age-specific respiratory minute volumes proportionally, taking into consideration the increased metabolic demand for this level of activity. To establish the activity-adjusted respiratory minute volume in all investigated age groups, half of the intensification was considered by a rise of the tidal volume and half by an acceleration of the breathing rate, as markup to the resting state (Figure 2B). 

The resting state tidal volume was established by a weight-adjusted physiological rate of 7 mL/kg [35,36], resulting in 103.6 mL for the 3y-child, 185.5 mL for the 8y-child and 500.5 mL for the 21y-adult. The resting state breathing frequency was adopted from literature with 26, 21 and 14 breaths per minute [33,37]. Table 1 provides the MPPD input parameters used:

To generate the VLP deposition heatmap for the size range between 0.3 µm and 100 µm, a series of calculations and modeling steps was performed. In a first step, a VLP exposure dose by mass was defined and converted into an exposure dose by particle number. Second, MPPD was applied to determine the VLP deposition per lung generation. Finally, the data were used to calculate a VLP deposition probability for a single inhaled VLP in each thoracic sub-region in relation to the total inhaled particles.

### 2.3. Deposition Modeling of Vocalization—And Breathing—Generated VLPs

Morawska et al. [22] and Johnson et al. [21] first experimentally established size distributions for respiratory aerosol particles originating from specific human expiratory activities. Our respiratory aerosol particle modeling is based on these data, which are broadly confirmed by findings of other research groups [38]. 

In short, to generate the data, the authors of both studies combined an expiratory droplet investigation system (EDIS) that includes an aerodynamic particle sizer (APS) to measure particles from 0.5 to 20 µm and a droplet deposition analysis (DDA) to measure particles larger than 20 µm. EDIS implements a wind tunnel system with an opening into which healthy volunteers place their heads and execute defined respiratory maneuvers on demand. The observed size distribution results were assumed to be a superposition of log-normal distributions, representing the specific respiratory maneuvers and anatomical sites of origin. Five distinct particle size distributions with associated count median diameters (CMD 1–5), were identified by curve fitting and assigned to expiratory maneuvers and anatomical origins. In our study, these five CMDs, as representatives for their particular log-normal size distributions, were applied for the in silico investigation of particle deposition characteristics (Table 2). 

### 2.4. Compilation into Regionalized Deposition Probability and Relative Inoculum Quantum Heatmaps

A three-tier approach was used to establish different levels of heatmaps, revealing lung lobe- and lung generations-specific VLP deposition probabilities (regionalization). A relative quantum heatmap was established to compare the age groups’ VLP deposition performance upon ambient air virion-laden aerosol exposure, originating from breathing and vocalization activity. 

First, MPPD was used to determine the VLP deposition probability in the thoracic region, per lung generation, particle CMD 1–4 and age group. VLP deposition probability heatmaps were established, based on the total inhaled particle number. CMD 5 was excluded from further analysis, because initial results revealed negligible contribution of these particles to thoracic deposition.

Second, weighted VLP deposition heatmaps were established by considering the relative abundance of CMD 1–5 VLPs in the exhalation plume and ambient air (Table 2). This step aimed at exploring selectivity for and differences in regional thoracic deposition patterns between investigated age.

Third, we compiled relative inoculum quantum heatmaps by normalizing the weighted VLP deposition data with the respiratory minute volume (breathing frequency x tidal volume; Table 1) of investigated age groups. Adult’s respiratory minutes volume was set as reference. These heatmaps represent the relative risk of an inoculating quantum upon identical exposure conditions, time span and VLP dose, for all three age groups. 

## 3. Results

A top-down approach was applied to elucidate differences between the three age groups in regard of regional thoracic VLP deposition preferences. For this study, we adopt the convention that VLPs in the size range below 5 µm are termed aerosols, bigger sizes are referred to as droplets; both being in equilibrium with ambient air. The combined impact of lung morphometry, breathing pattern, and aerosol characteristics were investigated at four different levels.

**Figure 3:**
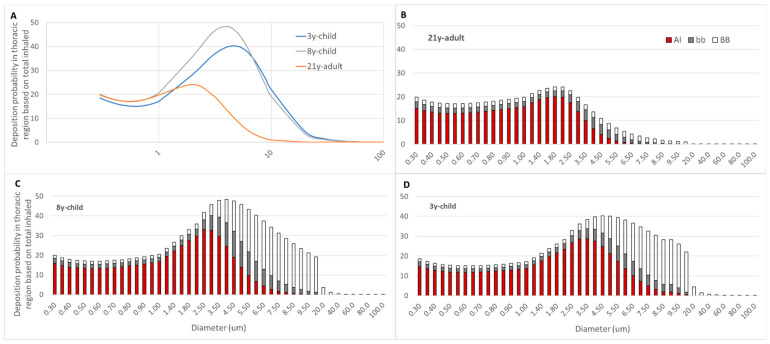
Thoracic deposition probability scan of VLPs in the size range of 0.3–100 µm for the three age groups, (**A**) cumulative (**B**–**D**) dissected in bronchial (BB), bronchiolar (bb) and alveolar–interstitial (AI) fractions. (**B**) 21y-adult, (**C**) 8y-child, (**D**) 3y-child. VLPs, respiratory virion-laden particles.

First, the thoracic deposition probability of single inhaled VLPs was established by scanning the size range of 0.3–100 µm for all three age groups. Figure 3A depicts the deposition probability chart for the three age groups. Considerable differences in deposition preferences and deposition fractions were observed. Both juvenile (3y-child, 8y-child) lung models unveiled markedly higher (more than 2-fold) peak VLP deposition probabilities compared to 21y-adult. This correlated with a prominent predilection to larger particle sizes, with a deposition probability maximum at 5.0 µm and 4.0 µm, respectively. 

A second, more detailed approach was then performed to generate insight in regionalized thoracic VLP deposition. This was achieved by dissecting the probability data into the BB, bb and AI compartment of each age group. Figure 3B–D depicts this view and instantly revealed the considerable preference for VLP deposition in the BB and bb region in both juvenile lung morphometry models, for particle sizes exceeding 2.5 µm. This was in significant contrast to the adult where the overall particle deposition probability peaked at 2.0 μm and only minor deposition of particles in the BB and bb region beyond this size threshold was observed. 

**Figure 4:**
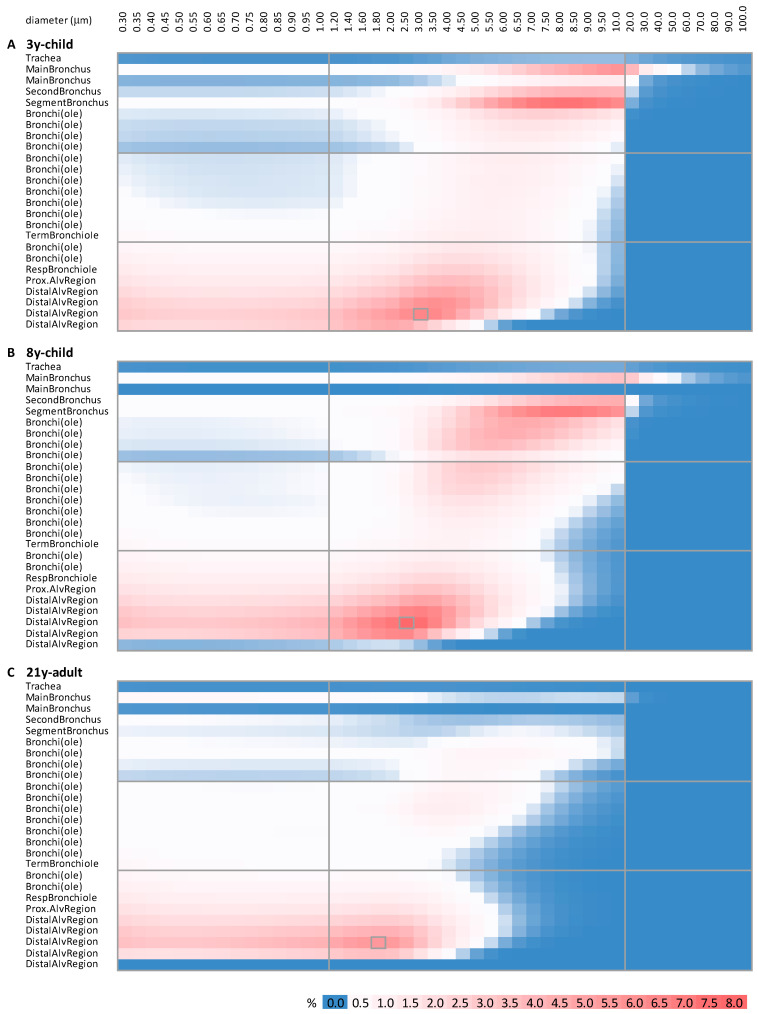
LRT deposition probability heatmaps, screening 0.3–100 µm and resolved in lung generations. Horizontal lines separate BB, bb and AI region, vertical lines VLP size ranges from 0.3–1 µm, >1 µm and >10 µm. Peak values in hotspots in AI regions are marked with grey boxes. Probability is color-coded according to legend. Heatmaps are shown for (**A**) 3y-child, (**B**) 8y-child and (**C**) 21y-adult. LRT, lower respiratory tract; BB, bronchial; bb, bronchiolar; AI, alveolar-interstitial.

A further level of refinement was made for the determination of regionalized deposition probability peaks and assigning these clusters of preferred deposition to anatomical regions of the lungs, from most central to most distal structures. For this purpose, we applied the detailed lung generation model presented in Figure 1. Remarkably, the children had two deposition probability peaks, one in the peripheral lung, at the third-last generation, associated with particle sizes of 3.0 µm (3y-child) and 2.5 µm (8y-child); the second peak in the BB, related to particle sizes determined at 10 μm (Figure 4A,B). The 21y-adult had a probability peak at 1.8 µm right there and no deposition peaks in the bb and BB region (Figure 4C). VLPs in the size range starting at 10 μm demonstrated negligible deposition probabilities for all age groups and all lung generations (Figure 4A–C).

**Figure 5:**
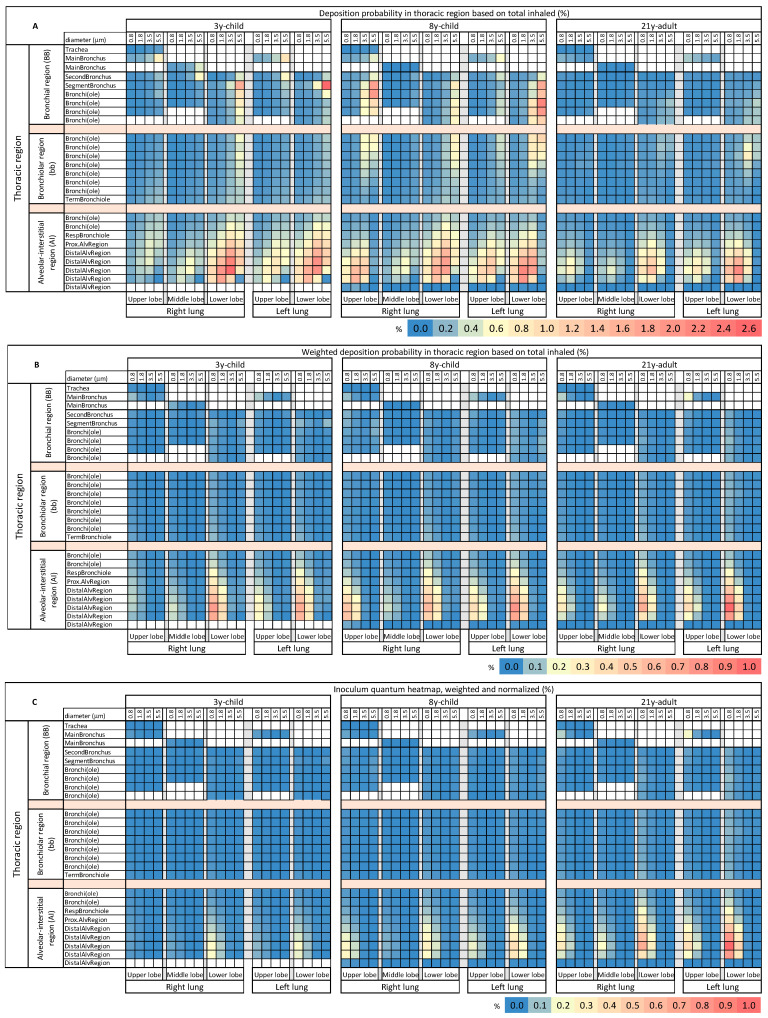
Lobe-specific LRT deposition probability heatmaps based on the number of total inhaled (3y-child, 8y-child, 21y-adult) particles. For lobe-specific analysis, four count median diameters diameter (CMD 1–4) for expiration activity vocalization and breathing (0.8 µm, 1.8 µm, 3.5 µm, 5.5 µm) were considered. As lobes have different numbers of generations, the sub-structures were aligned. Common structures are indicated on the far left. Probability is color-coded according to legend. Probability of deposition is depicted (**A**) unweighted, (**B**) weighted by relative abundance in the exhalation plume (CMD 1–4: 72.8%, 21.0%, 2.2%, 3.4%) and (**C**) as inoculum quantum heatmap, compensated for age-specific respiratory minutes volume. LRT, lower respiratory tract; CMD, count median diameter.

Finally, we applied our modeling to the breathing and vocalization activity-originating VLPs presented in Table 2, to mimic an indoor exposure of susceptible individuals to virion-laden aerosols. To generate lung lobe-specific VLP deposition heatmaps, we compiled our deposition probability data into up to 26 generations per lobe. Only four CMDs (CMD 1–4, 0.8 µm, 1.8 µm, 3.5 µm, 5.5 µm) were considered, CMD 5 (72.5 µm) was omitted due to the revealed neglectable probability for LRT deposition in all investigated age groups (Figure 4). Our lobe-specific LRT deposition probability heatmaps immediately unveiled deposition hotspots on both lower lobes in the AI region for all age groups (Figure 5A). Compared to adults (21y-adult), children (3y-child, 8y-child) showed a tendency for increased deposition probability of bigger-sized particle fractions in the AI hotspots; with a maximum for the 3.5 µm-sized particles, in all 5 lobes. Of note, additional hotspots in the BB region for larger aerosols (5.5 µm) in the lower lobes emerged in children. 

To improve the coherence of our modeling with actuality, we weighted the lobe-specific LRT deposition probability values by the relative abundance of the different size classes in the exhalation plume (Table 2). Remarkably, this abundance correction particularly reduced the intensity and magnitude of hotspots in the heatmaps for children (3y-child, 8y-child) in the AI region and eliminated the hotspots in the BB region, due to the minor abundance of the 3.5 and 5.5 µm-sized particles. The hotspots in the AI region translocated to the smallest particle sizes (0.8 µm) for all ages. Of note, the corrected deposition probabilities in the hotspots of the adult were higher than those of the children, equivalent to a lower susceptibility for virion-laden aerosol deposition in children’s peripheral lungs (Figure 5B). 

To enable a risk estimation for and comparison of direct inoculation and immediate disease initiation in the lower respiratory tract by virion-laden VLPs, we established regionalized inoculum quantum heatmaps. This was achieved by normalizing the weighted deposition probability data by the age-specific respiratory minute volume. The inoculum quantum heatmaps revealed that children, when compared to the adult as reference, were void of inoculum hotspots in the peripheral lungs, which were markedly present in the distal region of both lower lobes of the adult (Figure 5C). Significant inoculation in the central thoracic regions, in BB and bb, was not observed for any age group. Probability values and MPPD-derived raw data are available at https://doi.org/10.5281/zenodo.5543676, accessed on 1 October 2021.

## 4. Discussion

Our modeling approach focuses on elucidating age-specific disparities in the LRT regional inoculation with SARS-CoV-2-laden aerosols and droplets, thus, explaining differences in the frequency of LRT disease in children and adults and being causal to age-specific variants in COVID-19 pathogenesis. Such differences are expected due to the interplay of age-specific lung morphometry, respiratory patterns, and the physiochemical properties of VLPs determining their particokinetics. These effects may be confounded with differences in the barrier function of age-specific afflicted respiratory epithelia and tissue-specific capabilities of the initial immune defense. Moreover, age-specific and tissue-specific variability in the gene expression of virial docking receptors such as ACE2 and TMPRSS2 may also contribute. 

Age-related differences in the ability for self-aspiration of URT originating VLPs could hamper SARS-CoV-2 propagation to the LRT, thus, explain the in vivo observed lower rate of pulmonary involvement in children. The key parameter determining the potential for translocation of VLPs is the residual URT volume, in which aerosol particles in the oral cavity or nasal passages at the end of the expiratory phase are trapped and then immediately aspirated at the beginning of the next inhalation cycle. Although children have a lower URT volume than adults, they have a higher respiratory rate and a higher fraction of translocated residual URT volume within their total respiratory minute volume (URT volume min^−1^/respiratory volume min^−1^: 0.075 for 3y-child; 0.061 for 21y-adult; Table 1). Hence, the potential for aspiration in children is higher than in adults and cannot explain the lower rate of pulmonary involvement in vivo or give rise for the absence of hotspots in the peripheral lung in silico.

Our data on VLPs’ deposition probabilities revealed a markedly increased overall retention in the juvenile respiratory tract, being selectively driven by particles in the size range peaking at 4–5 µm. Based on the principles of particokinetics, the deposition of VLPs in this size class is dominated by impaction, favored by high flow rates associated with a juvenile respiratory tract morphometry. Counter-intuitively, the comparison of the 3y-child and 8y-child unveiled a higher deposition probability peak for the older. However, juvenile lung development is not a linear process. Alveolarization in the AI region and airway elongation in the BB and bb regions are not proportionally connected; the elongation of central airway structures favors retention of bigger-sized VLPs by impaction, alveolarization increases the deposition probability in the peripheral region by diffusion. The children’s overall higher deposition rate of micron-sized aerosol particles is well aligned with reported higher susceptibility of children to toxic particulate matter [11,12,13], which is furthermore enhanced by higher respiratory minute volumes relative to the body mass. The observed differences suggest a vulnerability of the juvenile respiratory tract for deposition and SARS-CoV-2 transmission by droplets, rather than virion-laden aerosols. A similar strong selectivity was not identified in the 21y-adult, which displayed a rather constant VLP retention for sizes up to 2 µm. 

Our regionalized LRT deposition probability heatmaps reveal three things: (i) VLPs with a size larger than 10 µm are negligible for thoracic SARS-CoV-2 inoculation, irrespective of age, which was not shown before for a juvenile lung morphometry. Below this size threshold, there are two major age-specific disparities in locality and intensity of bronchial and alveolar high VLP deposition areas. (ii) The heatmaps confirm deposition hotspots in the BB region of the juvenile lung, with no counterparts in that of the young adult. These hotspots are in the generations represented by and adjacent to segment bronchi in the BB region, involving VLPs in the size range of 4 up to 10 µm, which, however, are scarcely available in the exhalation plume. (iii) Marked deposition probability hotspots, present in all age groups, can be identified in the AI region, starting at the proximal alveolar region and intensifying towards the penultimate distal alveolar generation, representing the secondary pulmonary lobules. There is again an age-dependent trend of hotspot relocation towards bigger VLP sizes with decreasing age. The identification of these hotspots merits a more thorough investigation, because successful inoculation is not hampered by effective clearance by ciliated epithelial cells or via abundant macrophages. A deposition of SARS-CoV-2 virions on ciliated airway epithelia goes along with a markedly reduced probability for swift spike-driven virus-cell-fusion and successful replication, hindered by a high-viscous biphasic mucus layer, which has to be surmounted via stochastic, undirected diffusional translocation, and by effective mucociliary clearance with a velocity exceeding 1 mm/min in the BB region by far [39]. In addition, the alveolar fluid lining, a thin (average 200 nm) [40] aqueous hypophase with overlying surfactant film of low viscosity, allows diffusional translocation of virions onto alveolar epithelial cells without delay, thus, marking these hotspots as preferential anatomical sites for disease initiation.

To further elaborate the peculiarities of the identified LRT deposition hotspots, data were mapped to all five lung lobes and then weighted by the relative abundance of VLPs of respective sizes in the exhalation plume. The analysis revealed that all age groups were completely void of significant VLP deposition in the BB or bb region and implied a striking tendency of both lower lung lobes to be the most afflicted lung compartments with VLP deposition, associated with a dominant age-dependent intensity, highest in the 21y-adult and lowest in the 3y-child. This is a further piece of evidence for lower vulnerability of the juvenile lungs for disease initiation by aerosol transmission. Our observed selectivity for the AI region of both lower lobes is of major importance, because it is well aligned with the spatiotemporal distribution preferences of chest CT anomalies in adult COVID-19 patients with LRT involvement. The predominant radiologic anomalies in early stages of pulmonary involvement was the prominent peripheral distribution of ground-glass opacity (GGO), with subpleural localization [41], and predilection to both lower lobes [42]. GGO is described as a locally confined, delineated hazy increase in attenuation of secondary pulmonary lobules. These structures, comprised by units of three to five terminal bronchioles, roughly 200 in number in the AI region of both lungs. The low number of GGOs in early disease phase suggests very focal and limited VLP inoculation; however, sufficient for initiation of locally confined inflammation, partial filling and collapse of alveoli, interstitial thickening and increased capillary blood flow [43]. Hence, disease initiation right there is directly linked to clinical symptoms of pneumonia, dyspnea, and reduced blood oxygen saturation [44], thus, more severe disease. 

Our data on age-specific LRT inoculum quantum heatmaps reflects the relative LRT inoculum risk by considering the respiratory minute volume. This perspective unveils, for the first time, that children are nearly void of inoculum quantum aggregation in the AI region and elsewhere in the LRT, and that this is due to a combined effect of age-specific lung morphometry, respiratory pattern, and VLPs’ particularities. This was not different for our investigated aerosol when considering solely mouth breathing (MPPD breathing scenario “oral”), a scenario which would reflect the situation of a person with nasal airways obstruction. Only minor changes in the deposition probability values were observed, consistent in all age groups. Raw data, deposition probability values and heatmaps for mouth breathing are available at https://doi.org/10.5281/zenodo.5543676, accessed on 1 October 2021.

**Figure 6:**
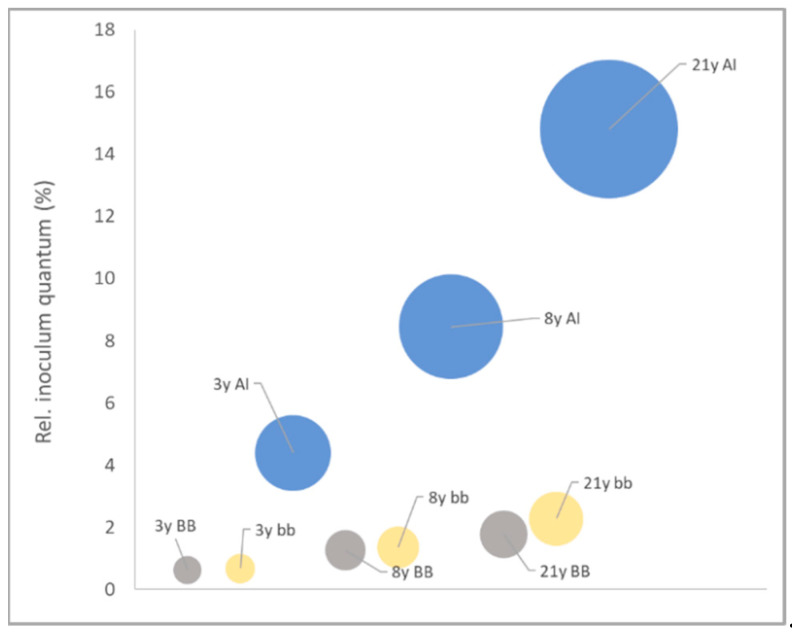
Age group-specific relative inoculum quantum for thoracic regions BB, bb and AI. Relative inoculum quantum ratios are represented by the differences in bubble area size. AI region is depicted in blue, bb region in yellow and BB region in grey. 3y, 3y-child; 8y, 8y-child; 21y, 21y-adult; BB, bronchial; bb, bronchiolar; AI, alveolar-interstitial.

The condensed lung generation data of inoculum quantum heatmaps reveals the particular role of the BB, bb and AI regions in age-specific initial virion deposition. Regardless of age, the BB and bb region demonstrate only a minor risk allocation for direct SARS-CoV-2 inoculation. However, the AI region instantly reveals its crucial role and compelling age correlation (Figure 6) for initial viral deposition and potential replication. 

At this point, the particular susceptibility of type II alveolar cells, which can be considered to be defenders of the alveolar homeostasis, and their role as the progenitor cells for type I pneumocytes in the AI region, is noteworthy [9]. Alveolar macrophage activation and neutrophil recruitment are first key events of innate immune activation in response to SARS-CoV-2 and the presence of alveolar damage-associated molecular patterns. A very recent study identified IP-10 (CXCL10) as key predictive cytokine for disease progression to severe courses of illness [45] with LRT involvement. Of note, IP-10 is a chemokine for recruitment of macrophages in response to viral invasion and tissue damage, but also acts as regulator of cell growth and proliferation [46]. However, in the absence of an adaptive immune response in the very early phase of alveolar SARS-CoV-2 infection, only a limited effect on curbing the viral replication can be expected by innate immunity. In the context of progressive tissue damage, alveolar epithelial repair by type II pneumocytes is crucial for the alveolar tissue restoration, and proliferative impairment due to SARS-CoV-2 infection may favor COVID-19 progression and disease severity by increasing loss of alveolar functionality. This may in particular contribute to the observed increased rate of acute respiratory distress syndrome in the elderly age groups [47]. 

Our data on inoculum quantum in the AI region proposes a risk ratio of roughly 1:4 (cumulative) and 1:5 (most afflicted lung generation in the lower lobes) between the 3y-child and the 21y-adult. This is well aligned with reported LRT clinical symptoms in 3.9% (29/742) [4] of children vs. 18.5% (338/1828) and 24.9% (493/1981) [48,49] reported shortness of breath in adults, confirming LRT involvement. A minimum infectious dose for SARS-CoV-2 is not established, but is a topic with decreasing ambiguity. Emerging deep sequence studies of the SARS-CoV-2 genome variability before and after the transmission bottleneck in infector–infectee pairs, report on bottleneck sizes of single digit virions, narrowing down the numbers of earlier investigations which concluded that 43 PFU correlated with 10% infected individuals [50] or proposed an infectious dose of 300 PFU [51] or more. A low disease-initiating dose may favor initial viral replication at anatomical regions with increased inoculum allocation, i.e., the AI region, as well as protect individuals with decreased inoculum aggregation, i.e., children, both arguments well supported by our data. 

The major limitation of our study is that the lung morphometry data used for the age group modeling do not reflect intraspecific variability. However, the elucidated disparities of the age group’s performance are very well marked and with robust age-correlated trends, likely offsetting the margin of error due to this model simplification.

## 5. Conclusions

Our data suggests that children’s juvenile respiratory physiology provides protection against effective aerosol SARS-CoV-2 inoculation in the LRT, which complements findings of Loske et al. reporting that children’s pre-activated antiviral innate immunity in the URT may effectively control early SARS-CoV-2 infection [5]. Although a limited URT infection prevents disease progression to the peripheral lung, limited aerosol inoculation in the LRT prevents disease initiation right there. Both mechanisms may co-found the low rate of modest to severe clinical presentations with pulmonary involvement in children. 

We provide evidence for the mechanism of age group-specific disparities of inoculation quantum allocation in the LRT upon virion-laden aerosol exposure. This is of important note, because virion-laden aerosol production in infected individuals strongly increases with pulmonary involvement [52], and in consequence would exclude asymptomatic children, void of LRT infection, from being highly contagious superspreaders. This may be particularly relevant, as the role of children in indoor environments (kindergarten, primary schools) within COVID-19, is politically laden and needs evidence-based discourse and positioning. 

## Figures and Tables

**Table 1:** MPPD parameter settings for breathing scenario “moderate to marked physical activity”.

Input Section	Scenario	Parameter	Value Setting		
			3y	8y	21y
Airway Morphometry	-	Model	Age-specific 5-Lobe		
		FRC (mL)	48.20	501.32	2123.75
		URT (mL)	9.47	21.03	42.27
Inhalant Properties	Aerosol	Density (g/cm^3^)	1.320–1.328 ^(a)^		
		Aspect Ratio	1.0 (= spherical)		
		Diameter (µm)	0.3–100.0 ^(b)^		
Exposure Condition	Constant Exposure	Body Orientation	Upright		
		Aerosol Concentration (mg/m^3^)	0.5 ^(c)^		
		Breathing Frequency (per min.)	31.6	27.1	19.3
		Tidal Volume (mL)	125.8	239.1	689.7
		Inspiratory Fraction	0.4		
		Pause Fraction	0.0		
		Breathing Scenario	Nasal		
		Deposition/Clearance	Deposition Only		

^(a)^ Above a particle size threshold of 0.7 µm, density is constant at 1.328 g/cm^3^. ^(b)^ One simulation per selected aerosol particle size. ^(c)^ concentration used (only relevant for calculating deposition probabilities and not intended to reflect real world exposure). FRC, functional residual capacity; URT, upper respiratory tract; 3y, 3y-child; 8y, 8y-child; 21y, 21y-adult.

**Table 2:** VLP characteristics for expiratory activity “breathing and vocalization”.

CMD *	Diameter (µm)	Expiratory Maneuver	Origin [7]	Relative Abundance in Exhalation Plume (%)	Density (g/cm^3^)
CMD 1	0.8	Breathing, vocalization	Bronchiolar fluid film burst	72.82	1.328
CMD 2	1.8	Vocalization	Laryngeal fluid film burst	20.98	1.328
CMD 3	3.5	Vocalization	Laryngeal fluid film burst	2.16	1.328
CMD 4	5.5	Vocalization	Laryngeal fluid film burst	3.40	1.328
CMD 5	72.5	Vocalization	Oral cavity	0.64	1.328

* count median diameter.

## Data Availability

The data presented in this study is publicly available online at https://doi.org/10.5281/zenodo.5543676, accessed on 1 October 2021.
